# Establishing defined daily doses (DDDs) for antimicrobial agents used in pigs, cattle and poultry in Japan and comparing them with European DDD values

**DOI:** 10.1371/journal.pone.0245105

**Published:** 2021-04-16

**Authors:** Kyoko Fujimoto, Mai Kawasaki, Reiko Abe, Takashi Yokoyama, Takeshi Haga, Katsuaki Sugiura

**Affiliations:** 1 Department of Global Agricultural Sciences, Graduate School of Agricultural and Life Sciences, The University of Tokyo, Tokyo, Japan; 2 Department of Veterinary Medical Sciences, Graduate School of Agricultural and Life Sciences, The University of Tokyo, Tokyo, Japan; Nitte University, INDIA

## Abstract

Monitoring of antimicrobial use is essential in the management of the development and selection of antimicrobial resistance. A variety of indicators has become available to monitor antimicrobial use in human and animal medicine. One of them is an indicator based on defined daily dose (DDD). By using the number of DDDs administered and normalising it by the population at risk of being treated over a defined period, one can estimate the number of treatment days with antimicrobial agents in a population. For veterinary medicine, the European Medicines Agency (EMA) has published the European values of DDD (DDDvet) for food-producing animals. In this study, we defined Japanese defined daily doses for antimicrobial agents (DDDjp) using DDD values that we previously assigned for antimicrobial products approved for use in pigs, cattle and poultry in Japan and compared them with DDDvet values. For the comparison, the quotient of Japanese and European values (QDDD) was calculated and the effect of the administration route and the number of active substances contained in the preparation was investigated. A total of 59 DDDjp values were defined for 43 antimicrobial agents using the data of 276 products approved for use in pigs. Likewise, a total of 55 DDDjp values were defined for 32 antimicrobial agents using the data of 196 products for use in cattle, and a total of 27 DDDjps values were defined for 25 antimicrobial agents using the data of 131 products approved for use in poultry. A comparison was made for 42, 28 and 17 pairs of DDDjp and DDDvet values for antimicrobial agents used for pigs, cattle and poultry respectively. The comparison showed median QDDD value of 0.61 and 0.66 for antimicrobial agents used for pigs and cattle respectively (*p*<0.01), indicating that the Japanese daily doses are significantly lower than the corresponding EMA values in these species. For the antimicrobial agents used for poultry, no significant difference was observed between DDDjp and DDDvet values with a median QDDD value of 1.15. The difference between DDDvet and DDDjp values and absence of DDDvet values for some antimicrobial agents marketed in Japan indicate that DDDjp rather than DDDvet should be used as the basis for the calculation of antimicrobial use monitoring in farm animals in Japan.

## Introduction

The use of antimicrobial agents in food-producing animals may lead to the emergence and selection of resistant bacteria. Bacterial resistance arises through complex mechanisms, including, in particular, mutation and selection, or by acquiring the genetic information that encodes resistance from other bacteria [1]. Therefore, reducing the selection pressure by reducing antimicrobial use is considered an important strategy to decrease resistance rate [1].

A valuable tool used to control and reduce antimicrobial use in veterinary medicine is the establishment of a monitoring system which can be created using various types of data collection methods [2]. In the EU, antimicrobial sales data are collected from each member country and antimicrobial consumption in each member is calculated and published in terms of milligrams of active ingredient sold per population correction unit (mg/PCU). The World Organisation for Animal Health (OIE: *Office International des Epizooties*) is attempting to develop a data collection system that enables the monitoring of antimicrobial use in each member country using a similar metric (mg of active ingredient per kg of animal biomass) [3]. The disadvantage of using these metrics is that the different potencies of different antimicrobial agents are not taken into account [4]. In human medicine, the World Health Organization (WHO) has determined an average daily maintenance dose as the main indication for each active substance [5]. The number of potential treatment days in a population can be estimated, using daily doses and the amount of an active ingredient administered. This statistical value has been adapted to veterinary medicine and is the basis of the national antibiotic monitoring systems in several Scandinavian countries and the Netherlands [6–8].

A list of defined daily doses for the food-producing animals (DDDvet) has been available from the European Medicines Agency (EMA) since 2016 [9]. Dose data from nine EU member states were collected and average values for the daily doses of each active ingredient by administration route (parenteral, oral except premix and premix) and by species were calculated. The active ingredients of each antimicrobial agent were classified based on anatomical-therapeutic-chemical correspondences (ATCvet Code) [10].

In Japan, veterinary antimicrobial products used for therapeutic purposes must be approved by the Minister of Agriculture, Forestry and Fisheries before they are manufactured and marketed for use; and they are permitted for use only when prescribed by veterinarians (no over-the-counter sales are permitted) [11]. Veterinary antimicrobial products must be used in line with the usage, dosage, withdrawal period and other conditions prescribed at the time of their approval by the Minister of Agriculture, Forestry and Fisheries [11]. The sales of antimicrobials for veterinary use are monitored under the Japanese Veterinary Antimicrobial Resistance Monitoring System (JVARM) established in 1999 [12]. Under this monitoring system, manufacturers and importers of veterinary antimicrobials are required to report annually to the Minister of Agriculture, Forestry and Fisheries on the quantity of veterinary antimicrobials sold for therapeutic use in cattle, pigs, poultry, dogs and cats. This monitoring process has revealed that between 600 and 700 tons of antimicrobials were sold annually for veterinary use. In addition, some antimicrobials (mostly ionophores such as monensin, salinomycin and narasin) are used as feed additives for growth promoting purposes without recourse to a prescription from a veterinarian. These antimicrobial feed additives are marketed after tested for quality by the Food and Agricultural Materials Inspection Center and used under the conditions prescribed by the Minister of Agriculture, Forestry and Fisheries [11]. In fiscal year 2019, a total of 75 tons of antimicrobial feed additives were tested and marketed [13]. Using these sales data and demographic data of food-producing animals in Japan, the authors have previously investigated the use of antimicrobial agents for therapeutic purposes in food-producing animals in Japan in terms of mg of active ingredient sold per kg of biomass and revealed that the annual use of veterinary antimicrobials in Japan remained 203–229 mg of active ingredient per kg of PCU between 2014 and 2017, which is relatively high compared with the usage in most European countries [14–16].

With the objective of establishing a monitoring system using an indicator based on daily dosage, the authors have recently assigned DDD values for 276, 196 and 131 veterinary antimicrobial products approved and marketed for use in Japan in pigs, cattle and poultry respectively [17, 18].

The aim of the present study was to define Japanese daily doses (DDDjp) for each antimicrobial agent (active ingredient) based on these DDD values assigned for products and to compare them with the EMA values.

## Materials and methods

### Defining Japanese DDD values for antimicrobial agents used for pigs, cattle and poultry in Japan (DDDjp)

The DDDjp values were calculated using the DDD values that the authors recently assigned for 276, 196 and 131 veterinary antimicrobial products approved and marketed in Japan for use in pigs, cattle and poultry, respectively [17, 18]. In this recent study, the DDD values for antimicrobial products (excluding intramammary and intrauterine products) were assigned by species and by kg of animal per day, using the principles developed by the European Medicines Agency (EMA) [19]. The DDD values for intramammary products for lactating cows and intrauterine products were also assigned by kg of animal per day using a standard body weight of 635kg [20] (see S2 Table for details).

The DDD value for a topical product (kanamycin sulfate for intranasal spray) was assigned by dividing the daily dose per administration by 3kg (standard weight of suckling piglet assigned, based on the average weight of suckling piglet [21]) and a long-acting factor of seven days [18].

In the current study, the DDDjp values were calculated by averaging the DDD values of products if two or more products contain the same antimicrobial agent, as follows:

$$DDDjp\;for\;antimicrobial\;agent\;a(mg/kg)=\frac{\sum_{i=1}^{n}{DDD}_{i}}{n}$$

where DDD*i* is the DDD value (mg/kg) of antimicrobial product *i* containing antimicrobial agent *a*, and *n* is the number of products containing antimicrobial agent *a*. For those antimicrobial agents that are used as the active ingredient in products for two or more administration routes, DDD values were assigned separately according to administration route. Likewise, for those that are used both in single substance and combination products, DDD values were assigned separately by the number of active ingredients contained in the preparation in the same way as the EMA, considering the synergistic action of the ingredients in combination products. In other words, applying the principles developed by the EMA [19], the average (arithmetic mean) of all DDD values of products for each combination of antimicrobial agent, administration route and the number of substances in the product (single substance or combination product) for each animal species was used to assign DDDjp–e.g, benzylpenicillin/injectable/single substance/pigs.

### Comparison of DDDjp and DDDvet values

To perform the comparison with the values of the EMA, the quotient of the daily doses (QDDD) was formed from Japanese and EMA values, as follows:

$$QDDD=\frac{DDDjp}{DDDvet}$$

If the quotient gives a value of one, it means that the Japanese dose and the EMA dose are the same. Quotients that exceed the value of one mean that the Japanese dosages are higher and values below one indicate that they are lower. If there was no EMA value for an active ingredient, the antimicrobial agent was excluded from the comparison. The effects on the comparison of the number of active ingredients contained in the product and the administration route was examined.

The comparison of DDDjp and DDDvet was not made for antimicrobials for intramammary and intrauterine administration routes because DDDvet values for these antimicrobials were not available per kg per day. The EMA defines DDDvet values for these antimicrobials on one unit per teat, udder or animal basis [9].

### Statistical analysis

The statistical analysis was performed to verify a deviation of QDDD between administration routes and number of active ingredients using R Statistical Software (version 4.0.3; R Foundation for Statistical Computing, Vienna, Austria). A *p*-value ≤ 0.05 was set as the significance level. The data were checked for normal distribution by Shapiro tests. The difference between DDDvet and DDDjp values was examined using the Wilcoxon test for paired samples. The effects of the administration routes and the number of active ingredients in the product were examined using a Mann-Whitney U test.

## Results

### Distribution of antimicrobial products approved for use in food-producing animals in Japan

A total of 59 DDDjp values were defined for 44 antimicrobial agents using the data of 276 products approved for use in pigs. Likewise, a total of 55 DDDjp values were defined for 32 antimicrobial agents using the data of 196 products for use in cattle. A total of 27 DDDjps values were defined for 25 antimicrobial agents using the data of 131 products approved for use in poultry. The distributions of the products according to administration route and number of active substance in the preparation are presented in Tables 1, 2 and 3. A complete list of the antimicrobials for which DDDjp values were defined are given in S1, S2 and S3 Tables.

**Table 1:** Distribution of antimicrobial products approved for use in pigs in Japan by administration route and number of active ingredient contained.

Product type	Administration route			Total
	Injection	Oral	Intranasal	
Single substance	80	135	1	216
Combination	18	42	0	60
Total	98	177	1	276

**Table 2:** Distribution of antimicrobial products approved for use in cattle in Japan by administration route and number of active ingredients contained.

Product type	Administration route				Total
	Injection	Oral	Intrauterine	Intramammary	
Single substance	96	48	0	18	162
Combination	18	6	2	8	34
Total	114	54	2	26	196

**Table 3:** Distribution of the antimicrobial products approved for use in poultry in Japan by administration route and the number of active ingredients contained.

Product type	Administration route		Total
	Injection	Oral	
Single substance	18	87	105
Combination	0	26	26
Total	18	113	131

### Comparison of the DDDjp values with DDDvet values for antimicrobial agens for use in pigs

**Fig 1:**
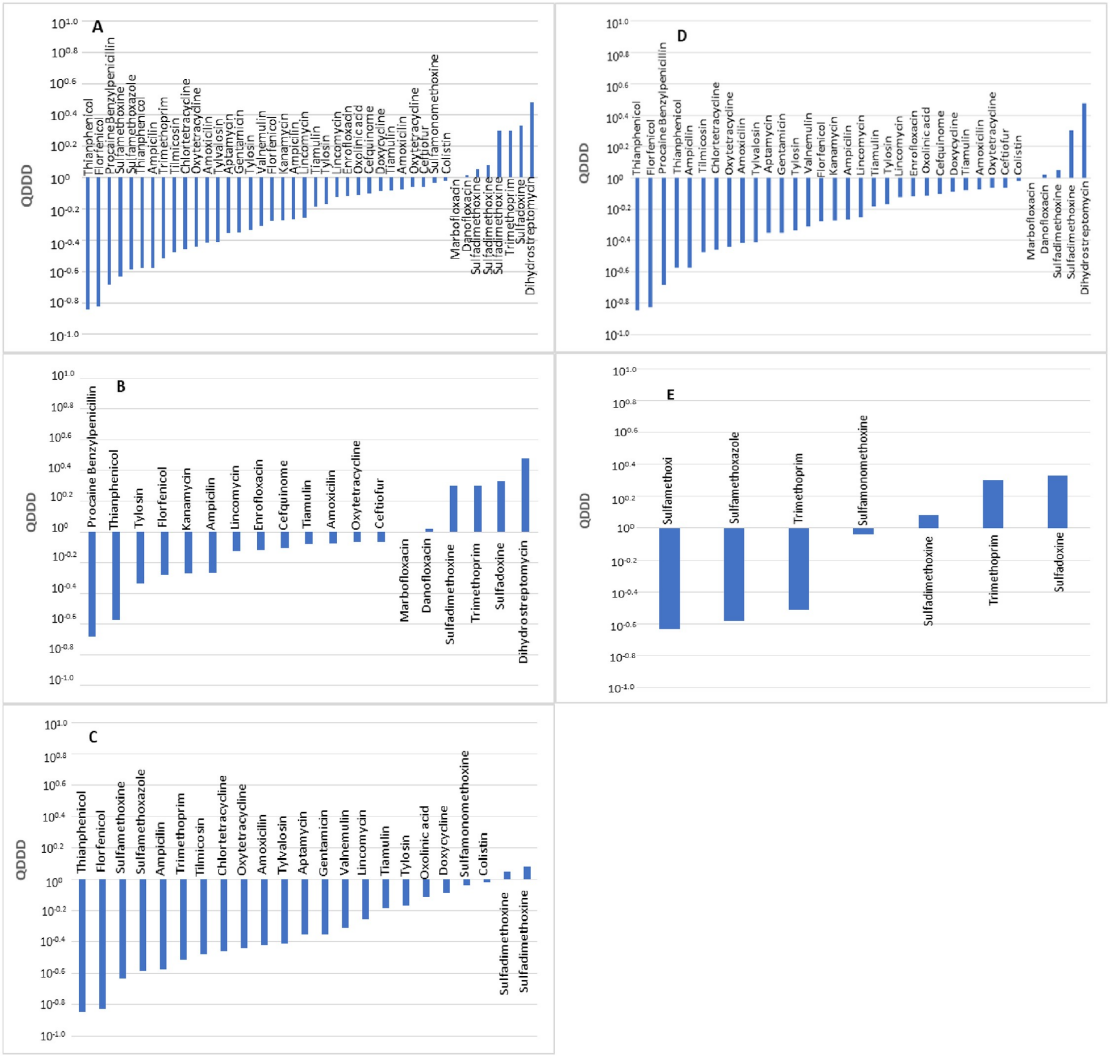
Comparison of the defined daily doses in Japan (DDDjp) of antimicrobial agents approved for use in pigs with the corresponding values of the European Medicines Agency (DDDvet). Bars indicate an antimicrobial agent for which both DDDjp and European values of DDD (DDDvet) values are available with QDDD (DDDjp/DDDvet) values in ascending order. Comparison of all antimicrobials (A), antimicrobial agents for injection (B), antimicrobial agents for oral administration (C), antimicrobial agent used in single substances (D) and antimicrobial agents used in combination products (E).

A comparison of 42 pairs of DDDjp and DDDvet values of antimicrobial agents for use in pigs was made. The distribution of the quotients of daily doses is shown in Fig 1. A total of 37 pairs revealed the DDDjp value to be lower than the DDDvet value. A total of 27 values compared showed deviations that exceeded 50%. A significant difference between the DDDvet and DDDjp values was observed (*p* <0.01) with a median QDDD value of 0.61.

The administration route revealed a significant difference (*p* = 0.007) in terms of the level of the deviations between the DDDjp and DDDvet values (Table 4). The five most frequently approved antimicrobial agents for each administration route are presented in Table 6. In comparison with the guideline values of the EMA, the injection solutions with the active substances kanamycin and ampicillin revealed lower daily doses with QDDD of 0.54. An injection suspension with the active substance procaine benzylpenicillin gave lower daily doses (QDDD = 0.21). Antimicrobial agents for oral administration (florfenicol and trimethoprim) were approved in Japan with lower daily doses (florfenicol: QDDD = 0.15, trimethoprim: QDDD = 0.31) (Table 5).

**Table 4:** Statistical evaluation of the calculated quotients QDDD of antimicrobial agents for use in pigs in Japan in relation to administration routes and the number of active ingredients contained in the preparation (Mann-Whitney U Test).

	Median QDDD	Statistical significance
Administration route		
Injection	0.833	Significant (*p* = 0.007)
Oral	0.444	
Number of substances		
Single	0.557	Not significant (*p* = 0.555)
Combination	0.917	

DDD defined daily doses

Median QDDD calculated quotient of Japanese daily doses and the corresponding values of the European Medicines Agency

**Table 5:** Average daily doses (DDDjp) of the most frequently approved antimicrobial agents (divided by administration routes) for use in pigs in Japan and their comparison with the values of the European Medicines Agency (DDDvet) based on calculated quotients (QDDD).

Administration route	Antimicrobial agent (active ingredient)	Number of substances	DDDjp (Number of products approved)	DDDvet	QDDD
Injection	Kanamycin	Single	15.0 (11)	28.0	0.54
	Procaine benzylpenicillin	Single	2.7 (8)	13.0	0.21
	Enrofloxacin	Single	2.6 (8)	3.4	0.76
	Oxytetracycline	Single	6.5 (7)	7.5	0.87
	Ampicillin	Single	6.5 (7)	12.0	0.54
Oral	Florfenicol	Single	1.5 (18)	10.0	0.15
	Tiamulin	Single	6.4 (15)	9.7	0.66
	Tylosin	Single	8.1 (13)	12.0	0.68
	Doxycycline	Single	9.0 (10)	11.0	0.82
	Trimethoprim	Combination	1.4 (9)	4.7	0.31

DDDjp defined daily doses of the active ingredient used in pigs in Japan established in this study.

DDDvet defined daily doses of the active ingredient used in pigs in Europe established by the European Medicines Agency

QDDD calculated quotient of DDDjp/DDDvet

The number of active substances contained in a veterinary medicinal product (*p* <0.01) did not reveal a significant difference in terms of the level of the deviations between the daily doses DDDjp and DDDvet (Table 4).

### Comparison of the DDDjp values with DDDvet values for antimicrobial agents for use in cattle

**Fig 2:**
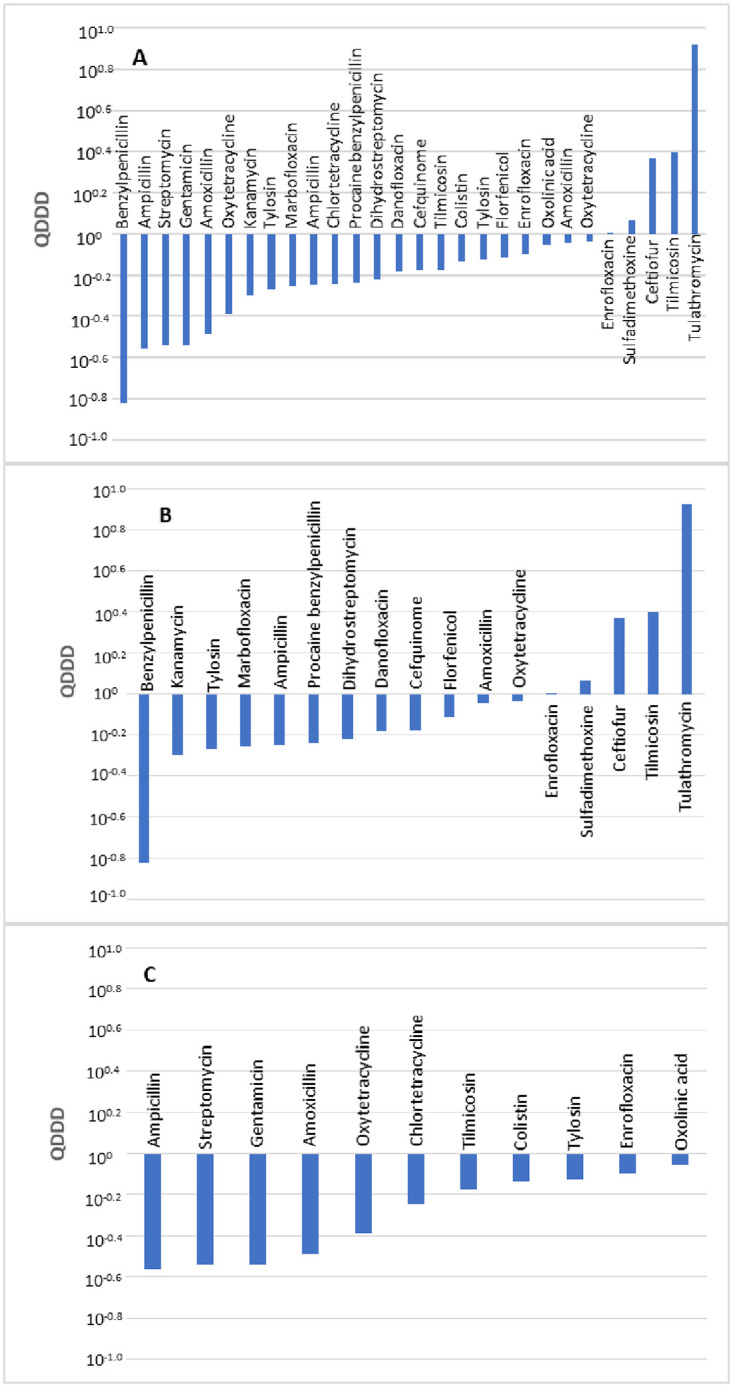
Comparison of the defined daily doses in Japan (DDDjp) of antimicrobial agents approved for use in cattle with the corresponding values of the European Medicines Agency (DDDvet). Bars indicate an antimicrobial agent for which both DDDjp and European values of DDD (DDDvet) values are available with QDDD (DDDjp/DDDvet) values in ascending order. Comparison of all antimicrobials (A), antimicrobial agents for injection (B) and antimicrobial agents for oral administration (C). Comparison was not made by number of substances because there was no combination antimicrobial agent for which DDDvet and DDDjp values were available.

A comparison of 27 pairs of DDDjp and DDDvet values of antimicrobial agents for injection and oral use in cattle was made. A comparison was not made for antimicrobial agents for intramammary and intrauterine use because DDDvet values were not available for antimicrobial agents for these administration routes. The distribution of the quotients of daily doses is shown in Fig 2. A total of 10 compared values showed deviations of over 50%. A significant difference between the DDDvet and DDDjp values was observed for antimicrobial agents used in cattle (*p* <0.01) with a median QDDD value of 0.66.

Neither the administration route nor the number of active substances contained in a veterinary medicinal product revealed a significant difference in terms of level of deviations between the DDDjp and DDDvet values (Table 6). In comparison with the EMA guideline values, the injection solutions with active substances ampicillin, kanamycin and procaine benzylpenicillin showed lower daily doses with QDDD of 0.56, 0.50 and 0.58, respectively. Antimicrobial agents for oral administration (ampicillin, amoxillin and oxytetracycline) were approved in Japan with lower daily doses with QDDD of 0.28, 0.33 and 0.41 respectively (Table 7).

**Table 6:** Statistical evaluation of calculated quotients QDDD of antimicrobial agents for use in cattle in Japan in relation to administration route and number of active ingredients contained in the preparation (Mann-Whitney U Test).

	Median QDDD	Statistical significance
Administration route		
Injection	0.667	Not significant (*p* = 0.086)
Oral	0.568	
Number of substances		
Single	0.632	−*
Combination	−*	

DDD defined daily doses

Median QDDD calculated quotient of Japanese daily doses and the corresponding values of the European Medicines Agency

* Statistical evaluation was not possible because there was no combination antimicrobial agent for which DDDvet and DDDjp values were available

**Table 7:** Average daily doses (DDDjp) of the most frequently approved antimicrobial agents (divided by administration routes) for use in cattle in Japan and their comparison with the values of the European Medicines Agency (DDDvet) based on calculated quotients (QDDD).

Administration route	Antimicrobial agent (active ingredient)	Number of substances	DDDjp (number of products approved)	DDDvet	QDDD
Injection	Ampicillin	Single	6.2 (14)	11.0	0.56
	Kanamycin	Single	7.5 (11)	15.0	0.50
	Florfenicol	Single	10.0 (9)	13.0	0.77
	Procaine benzylpenicillin	Single	7.5 (8)	13.0	0.58
	Enrofloxacin	Single	4.2 (8)	4.2	1.00
Oral	Ampicillin	Single	8.0 (8)	29.0	0.28
	Amoxicillin	Single	6.5 (8)	20.0	0.33
	Oxytetracycline	Single	8.1 (7)	20.0	0.41
	Chlortetracycline	Single	12.5 (6)	22.0	0.57
	Oxolinic acid	Single	15.0 (4)	17.0	0.88

DDDjp defined daily doses of the active ingredient used in pigs in Japan established in this study.

DDDvet defined daily doses of the active ingredient used in pig in Europe established by the European Medicines Agency

QDDD calculated quotient of DDDjp/DDDvet

### Comparison of the DDDjp values with DDDvet values for antimicrobial agents for use in poultry

**Fig 3:**
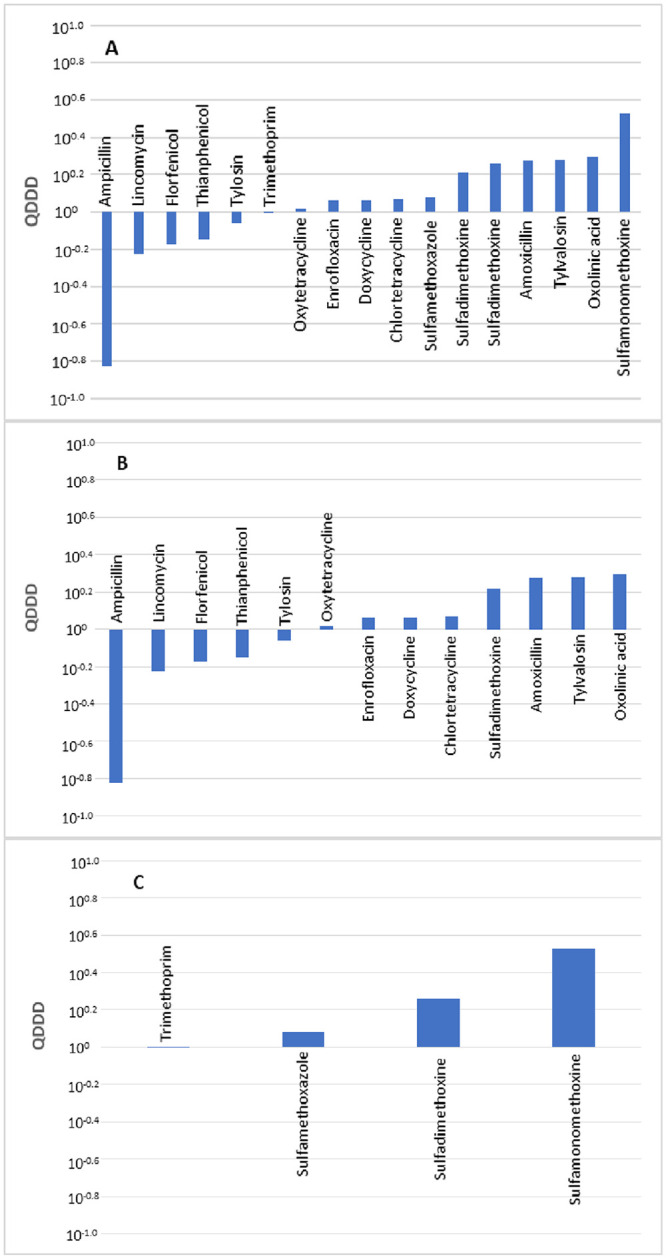
Comparison of the defined daily doses in Japan (DDDjp) of antimicrobial agents approved for use in poultry with the corresponding values of the European Medicines Agency (DDDvet). Bars indicate an antimicrobial agent for which both DDDjp and European values of DDD (DDDvet) values are available with QDDD (DDDjp/DDDvet) values in ascending order. Comparison of all antimicrobials (A), antimicrobial agent used in single substance products (B) and antimicrobial agents used in combination products (C). Comparison was not made by administration route because there was no antimicrobial agent for injection for which DDDvet and DDDjp values were available.

A comparison of 17 pairs of DDDjp and DDDvet values of antimicrobial agents for use in poultry was made. The distribution of the logarithmic quotients of daily and treatment dosages is presented in Fig 3. A total of five compared values resulted in deviations that exceeded 50%. No statistically significant difference was observed between the DDDvet and DDDjp values with a median QDDD value of 1.15.

Neither the administration route nor the number of active substances contained in a veterinary medicinal product revealed a significant difference in terms of the level of deviations between the DDDjp and DDDvet values (Table 8). When compared with the EMA guideline values, the oral solutions with the active substance ampicillin showed a lower daily dose with QDDD of 0.15 and amoxicillin with a higher daily dose with QDDD of 1.88 (Table 9).

**Table 8:** Statistical evaluation of the calculated quotients QDDD of antimicrobial agents for use in poultry in relation to administration routes and the number of active ingredients contained in the preparation (Mann-Whitney U Test).

	Median QDDD	Statistical Significance
Administration route		
Injection	−*	−*
Oral	1.153	
Number of substances		
Single	1.150	Not significant (*p* = 0.245)
Combination	1.508	

DDD Defined Daily Doses

Median QDDD calculated quotient of Japanese daily doses and the corresponding values of the European Medicines Agency

* Median QDDD value was not available and statistical evaluation was not possible because there was no antimicrobial agent for injection for which DDDvet and DDDjp values were available

**Table 9:** Average daily doses (DDDjp) of the most frequently approved antimicrobial agents for use in poulry in Japan and their comparison with the values of the European Medicines Agency (DDDvet) based on calculated quotients (QDDD).

Administration route	Antimicrobial agent (active ingredient)	Number of substances	DDDjp (Number of products approved)	DDDvet	QDDD
Oral	Tylosin	Single	78.9 (13)	81.0	0.97
	Doxycycline	Single	17.3 (12)	15.0	1.15
	Ampicillin	Single	16.3 (8)	108.0	0.15
	Amoxicillin	Single	30.0 (8)	16.0	1.88
	Oxytetracycline	Single	40.7 (7)	39.0	1.04

DDDjp defined daily doses of the active ingredient used in pigs in Japan established in this study.

DDDvet defined daily doses of the active ingredient used in pig in Europe established by the European Medicines Agency

QDDD calculated quotient of DDDjp/DDDvet

## Discussion

The present study defined national daily dosages (DDDjp) for the first time for all antimicrobial agents used in products approved for use in pigs, cattle and poultry in Japan. A comparison with corresponding values of the EMA was performed for most antimicrobial agents.

### Difference between DDDjp and DDDvet values

The comparison within this study shows that the medians of DDDjp and DDDvet values differ significantly, and that DDDjp values of some antimicrobial agents have considerable deviations from corresponding DDDvet values, e.g. the DDDjp value of 2.1 for procaine-penicillin/injection/single substance/pigs, which is more than 79% lower than DDDvet. Deviations have been observed in previous studies that compared the difference between DDDvet and DDD values of individual countries: A study conducted in Canada found that in developing their country-specific DDD values, the majority of their DDD values were lower than their corresponding DDDvet values [22]. In a study conducted by Echtermann defining Swiss daily doses (DDDch), the difference between DDDch and DDDvet values was not as significant as that between DDDjp and DDDvet values in the current study [23]. O’Neal at al. reported that by comparing the monitoring results using DDD values established by the EMA, Denmark and the Netherlands, different DDD systems produced different consumption patterns even though the underlying data for each was identical [24].

A comparison of DDDjps and DDDvet for antimicrobials destined for intramammary administration was not made in our study, because DDDvet values were not available in mg/kg/day. The DDDjp values for these antimicrobials were assigned in mg/kg/day assuming a standard weight at treatment and long-acting factor (for intramammary products for dry cows) to make them comparable with the DDD values of other countries defined in mg/kg/day (e.g., Canada [25]). However, the DDD values for dry cows are greatly affected depending on what values are used as a long-acting factor: we used a long-acting factor of four days in the same way as the Netherlands, whilst Germany and Canada used a long-acting factor of seven and ten days respectively, in assigning their DDD values for intramammary products for dry cows [25]. These periods are not representative of the exact true duration of action for each product but approximations and are defined strictly for allowing assignment of daily doses in a transparent way. To make an international comparison possible, a standard long-acting factor might be considered in assigning the DDD values for intramammary products for dry cows.

### Possible reasons for the difference between DDDjp and DDDvet values

There are many reasons for the difference observed between DDDvet and DDDjp or DDD values in other non-European countries. One reason is that the EMA might have had a wider range of antimicrobial doses to work with due to the collection of antimicrobial agent doses from nine European countries [9, 19]. The different labelling regulations, different treatment indications and different husbandry practices might all contribute to the variations in DDDvet and DDDjp values.

In Japan, veterinary antimicrobial products must be approved by the Minister of Agriculture, Forestry and Fisheries prior to manufacture and marketing for use [11]. Approval procedures of a new product even with an active ingredient used in previously approved products but with new dosages take more time and cost more than those with previously approved dosages; therefore it is common practice that approval application of new antimicrobial products containing an active ingredient that has been previously approved is performed with previously approved dosages. Thus, cases might occur in which dosages of antimicrobial agents used in the products that were first approved in the 1980s-1990s are not updated to reflect the dose needed to effectively and safely treat common veterinary pathogens. An outdated dosing problem has been reported in equine and bovine antimicrobial labelling in Australia [26, 27]. However, a further study is required to verify if this is the case in Japan. Fully elucidating the reasons for these differences is beyond the scope of this study. With the knowledge that underdosing can produce some of the worst possible effects on resistance selection, those products approved many years ago with very low dosages might require reevaluation to ensure the dosages are fully adapted to today’s resistance situation.

### Adequacy of using Japanese DDDs for the monitoring of antimicrobial use

This study showed that DDDvets did not cover all the antimicrobial agents used in veterinary medicine in Japan. Although drawing conclusions from differences between assigned DDDjp and DDDvet values is difficult, the difference between DDDvet and DDDjp values and absence of DDDvet values for some antimicrobial agents marketed in Japan indicate that DDDjp rather than DDDvet should be used as the basis for the calculation of antimicrobial use monitoring in farm animals in Japan, assuming that DDDjp better reflects the actual dosage used in food-producing animals in Japan. This is a reasonable assumption considering that Japanese veterinarians are more likely to follow dosage instructions rather than European instructions when they treat food-producing animals using antimicrobials marketed in Japan. To determine if the use of DDDjp can be recommended as the basis for the calculation of antimicrobial use on farms in Japan, the application of DDDjp and DDDvet values to calculate the numbers of DDDs for comparison using actual antimicrobial usage data on farms is indispensable, but this will be a subject of future studies.

In regard to standardized DDD values that should be used for international comparison, our previous report [28] revealed that there is a considerable difference in the number of DDDs when calculated using DDDjp and when calculated using DDDvet; this indicates that national DDDs do not enable an appropriate comparison with other countries, and a standard set of DDD values (such as the DDDvet) might be required.

## Supporting information

S1 Table Japanese DDD values (DDDjp) defined in this study for antimicrobial agents used in pigs in Japan and corresponding DDD values (DDDvet) defined by the European Medicines Agency. (DOCX)

S2 Table Japanese DDD values (DDDjp) defined in this study for antimicrobial agents used in cattle in Japan and corresponding DDD values (DDDvet) defined by the European Medicines Agency. (DOCX)

S3 Table Japanese DDD values (DDDjp) defined in this study for antimicrobial agents used in poultry in Japan and corresponding DDD values (DDDvet) defined by the European Medicines Agency. (DOCX)
