# Supplementary material for: Establishing defined daily doses (DDDs) for antimicrobial agents used in pigs, cattle and poultry in Japan and comparing them with European DDD values
Source: PLoS One. 2021 Apr 16;16(4):e0245105. doi: 10.1371/journal.pone.0245105 (PMC8051781; doi:10.1371/journal.pone.0245105)
Supplement: S1 Table — (DOCX) [file pone.0245105.s001.docx]

**S1 Table**

**Japanese DDD values (DDDjp) defined in this study for antimicrobial agents used in pigs in Japan and corresponding DDD values (DDDvet) defined by the European Medicines Agency**

| Antimicrobial class | Antimicrobial agent  (active ingredient) | Product type | Administration route | DDDvet  (mg/kg) | DDDjp  (mg/kg) | Number of products |
| --- | --- | --- | --- | --- | --- | --- |
| Tetracyclines | Oxytetracycline | Single substance | Injection | 7.5 | 6.5 | 2 |
| Amphenicols | Thiamphenicol | Single substance | Injection | 75.0 | 20.0 | 2 |
|  | Florfenicol | Single substance | Injection | 9.5 | 5.0 | 4 |
| Penicillins | Ampicillin | Single substance | Injection | 12.0 | 6.5 | 7 |
|  | Amoxicillin | Single substance | Injection | 8.9 | 7.5 | 1 |
|  | Procaine Benzylpenicillin | Single substance | Injection | 13.0 | 2.7 | 8 |
|  | Procaine Benzylpenicillin | Combination | Injection |  | 7.2 | 6 |
| Cephalosporins | Ceftiofur | Single substance | Injection | 3.0 | 2.6 | 5 |
|  | Cefquinome | Single substance | Injection | 1.9 | 1.5 | 2 |
|  | Sulfadimethoxine | Single substance | Injection | 30.0 | 60.0 | 2 |
| Sulfonamides | Sulfamonomethoxine | Single substance | Injection |  | 70.0 | 1 |
|  | Sulfadoxine | Combination | Injection | 14.0 | 30.0 | 3 |
| Trimethoprim | Trimethoprim | Combination | Injection | 3.0 | 6.0 | 3 |
| Macrolides | Tylosin | Single substance | Injection | 13.0 | 6.0 | 3 |
|  | Tulathromycin | Single substance | Injection |  | 2.5 | 4 |
| Lincosamides | Lincomycin | Single substance | Injection | 10.0 | 7.5 | 4 |
| Aminoglycosides | Dihydrostreptomycin | Single substance | Injection | 20.0 | 60.0 | 2 |
|  | Dihydrostreptomycin | Combination | Injection |  | 15.0 | 6 |
|  | Kanamycin | Single substance | Injection | 28.0 | 15.0 | 11 |
|  | Kanamycin | Single substance | Intranasal |  | 5.2 | 1 |
| Quinolones | Enrofloxacin | Single substance | Injection | 3.4 | 2.6 | 8 |
|  | Danofloxacin | Single substance | Injection | 1.2 | 1.3 | 1 |
|  | Marbofloxacin | Single substance | Injection | 2.0 | 2.0 | 3 |
|  | Orbifloxacin | Single substance | Injection |  | 3.8 | 3 |
| Pleuromutilins | Tiamulin | Single substance | Injection | 12.0 | 10.0 | 2 |
| Tetracyclines | Doxycycline | Single substance | Oral | 11.0 | 9.0 | 10 |
|  | Chlortetracycline | Single substance | Oral | 31.0 | 10.8 | 6 |
|  | Chlortetracycline | Combination | Oral |  | 6.0 | 2 |
|  | Oxytetracycline | Single substance | Oral | 26.0 | 9.4 | 7 |
|  | Oxytetracycline | Combination | Oral |  | 7.0 | 1 |
| Amphenicoles | Thianphenicol | Single substance | Oral | 35.0 | 5.0 | 7 |
|  | Florfenicol | Single substance | Oral | 10.0 | 1.5 | 18 |
| Penicillins | Ampicillin | Single substance | Oral | 30.0 | 8.0 | 8 |
|  | Amoxicillin | Single substance | Oral | 17.0 | 6.5 | 8 |
|  | Procain benzylpenicillin | Combination | Oral |  | 0.8 | 5 |
| Sulfonamides | Sulfadimethoxine | Single substance | Oral | 48.0 | 54.0 | 2 |
|  | Sulfadimethoxine | Combination | Oral | 24.0 | 28.8 | 2 |
|  | Sulfamonomethoxine | Single substance | Oral |  | 40.0 | 7 |
|  | Sulfamonomethoxine | Combination | Oral | 9.4 | 8.6 | 4 |
|  | Sulfamethoxazole | Combination | Oral | 20.0 | 4.7 | 7 |
|  | Sulfadimidine | Combination | Oral | 23.0 | 6.0 | 2 |
| Trimethoprims | Trimethoprim | Combination | Oral | 4.7 | 1.4 | 9 |
|  | Ormethoprim | Combination | Oral |  | 2.9 | 4 |
| Macrolides | Tylosin | Single substance | Oral | 12.0 | 11.3 | 13 |
|  | Tilmicosin | Single substance | Oral | 15.0 | 5.0 | 9 |
|  | Tylvalosin | Single substance | Oral | 3.6 | 1.4 | 2 |
|  | Mirosamycin | Single substance | Oral |  | 2.5 | 0 |
| Lincosamides | Lincomycin | Single substance | Oral | 7.6 | 4.2 | 6 |
| Aminoglycosides | Streptomycin | Single substance | Oral |  | 20.0 | 1 |
|  | Streptomycin | Combination | Oral |  | 4.2 | 3 |
|  | Gentamicin | Single substance | Oral | 1.4 | 0.6 | 1 |
|  | Kanamycin | Combination | Oral |  | 4.2 | 2 |
|  | Apramycin | Single substance | Oral | 9.0 | 4.0 | 1 |
|  | Fragiomycin | Combination | Oral |  | 4.9 | 1 |
| Fluoroquinolones | Norfloxacin | Single substance | Oral |  | 7.5 | 1 |
|  | Orbifloxacin | Single substance | Oral |  | 3.80 | 2 |
| Other quinolones | Oxolinic acid | Single substance | Oral | 26.0 | 20.0 | 3 |
|  | Tiamulin | Single substance | Oral | 9.7 | 6.4 | 15 |
| Pleuromutilins | Valnemulin | Single substance | Oral | 5.3 | 2.6 | 1 |
| Polymyxins | Colistin | Single substance | Oral | 5.0 | 4.8 | 7 |
| Total |  |  |  |  |  | 276 |

DDDvet DDD values in mg/kg/day defined by the European Medicines Agency (EMA)

DDDjp DDD values in mg/kg/day defined in this study using DDD values of antimicrobial products approved and marketed for use in Japan
