# Supplementary material for: Establishing defined daily doses (DDDs) for antimicrobial agents used in pigs, cattle and poultry in Japan and comparing them with European DDD values
Source: PLoS One. 2021 Apr 16;16(4):e0245105. doi: 10.1371/journal.pone.0245105 (PMC8051781; doi:10.1371/journal.pone.0245105)
Supplement: S2 Table — (DOCX) [file pone.0245105.s002.docx]

**S2 Table**

**Japanese DDD values (DDDjp) defined in this study for antimicrobial agents used in cattle in Japan and corresponding DDD values (DDDvet) defined by the European Medicines Agency**

| Antimicrobial class | Antimicrobial agent  (active ingredient) | Product type | Administration route | DDDvet  (mg/kg) | DDDjp  (mg/kg) | Number of products |
| --- | --- | --- | --- | --- | --- | --- |
| Tetracyclines | Oxytetracycline | Single substance | Injection | 6.5 | 6.0 | 7 |
| Amphenicols | Thiamphenicol | Single substance | Injection |  | 20.0 | 2 |
|  | Florfenicol | Single substance | Injection | 13.0 | 10.0 | 9 |
| Penicillins | Ampicillin | Single substance | Injection | 11.0 | 6.2 | 14 |
|  | Ampicillin | Combination | Injection |  | 5.0 | 3 |
|  | Amoxicillin | Single substance | Injection | 8.3 | 7.5 | 1 |
|  | Benzylpenicillin | Single substance | Injection | 14.0 | 2.1 | 1 |
|  | Cloxacillin | Combination | Injection |  | 5.0 | 3 |
|  | Procaine benzylpenicillin | Single substance | Injection | 13.0 | 7.5 | 8 |
|  | Procaine benzylpenicillin | Combination | Injection |  | 4.2 | 6 |
| Cephalosporins | Cefazolin | Single substance | Injection |  | 5.0 | 4 |
|  | Ceftiofur | Single substance | Injection | 1.0 | 2.3 | 5 |
|  | Cefquinome | Single substance | Injection | 1.5 | 1.0 | 2 |
| Sulfonamides | Sulfadimethoxine | Single substance | Injection | 30.0 | 35.0 | 2 |
|  | Sulfamonomethoxine | Single substance | Injection |  | 25.0 | 1 |
| Macrolides | Tylosin | Single substance | Injection | 13.0 | 7.0 | 3 |
|  | Tulathromycin | Single substance | Injection | 0.3 | 2.5 | 3 |
|  | Tilmicosin | Single substance | Injection | 4.0 | 10.0 | 5 |
| Aminoglycosides | Dihydrostreptomycin | Single substance | Injection | 25.0 | 15.0 | 2 |
|  | Dihydrostreptomycin | Combination | Injection |  | 8.8 | 6 |
|  | Kanamycin | Single substance | Injection | 15.0 | 7.5 | 11 |
| Quinolones | Enrofloxacin | Single substance | Injection | 4.2 | 4.2 | 8 |
|  | Danofloxacin | Single substance | Injection | 1.9 | 1.3 | 1 |
|  | Marbofloxacin | Single substance | Injection | 3.6 | 2.0 | 3 |
|  | Orbifloxacin | Single substance | Injection |  | 3.8 | 3 |
| Others | Fosfomycin | Single substance | Injection |  | 15.0 | 1 |
| Tetracyclines | Chlortetracycline | Single substance | Oral | 22.0 | 12.5 | 6 |
|  | Oxytetracycline | Single substance | Oral | 20.0 | 8.1 | 7 |
|  | Oxytetracycline | Combination | Oral |  | 12.5 | 1 |
| Penicillins | Ampicillin | Single substance | Oral | 29.0 | 8.0 | 8 |
|  | Amoxicillin | Single substance | Oral | 20.0 | 6.5 | 8 |
| Sulfonamides | Sulfamonomethoxine | Single substance | Oral |  | 45.0 | 7 |
|  | Sulfamonomethoxine | Combination | Oral |  | 11.3 | 2 |
| Trimethoprims | Ormethoprim | Combination | Oral |  | 3.8 | 2 |
| Macrolides | Tylosin | Single substance | Oral | 41.0 | 30.8 | 1 |
|  | Tilmicosin | Single substance | Oral | 21.0 | 14.1 | 2 |
| Aminoglycosides | Streptomycin | Single substance | Oral | 70.0 | 20.0 | 1 |
|  | Gentamicin | Single substance | Oral | 7.0 | 2.0 | 1 |
|  | Fragiomycin | Combination | Oral | 7.4 | 8.8 | 1 |
| Fluoroquinolones | Enrofloxacin | Single substance | Oral | 4.7 | 3.8 | 1 |
| Other quinolones | Oxolinic acid | Single substance | Oral | 17.0 | 15.0 | 3 |
| Polymyxins | Colistin | Single substance | Oral | 4.8 | 3.5 | 2 |
| Others | Fosfomycin | Single substance | Oral |  | 30.0 | 1 |
| Penicillins | Procaine bensylpenicillin | Combination | Intrauterine |  | 0.2 | 1 |
| Aminoglycosides | Dihydrostreptomycin | Combination | Intrauterine |  | 0.3 | 1 |
| Cephalosporins | Cefazolin | Single substance | Intramammary |  | 0.4 | 9 |
|  | Cefalonium | Single substance | Intramammary |  | 0.5 | 3 |
|  | Cefuroxime | Single substance | Intramammary |  | 0.6 | 2 |
| Tetracyclines | Oxytetracycline | Single substance | Intramammary |  | 1.1 | 1 |
| Penicillins | Procaine benzylpenicillin | Combination | Intramammary |  | 0.6 | 4 |
|  | Dicloxacillin | Single substance | Intramammary |  | 0.6 | 2 |
| Aminoglycosides | Dihydrostreptomycin | Combination | Intramammary |  | 1.0 | 2 |
|  | Kanamycin | Combination | Intramammary |  | 0.5 | 1 |
|  | Fradiomycin | Combination | Intramammary |  | 0.5 | 1 |
| Lincosamides | Pirlimycin | Single substance | Intramammary |  | 0.1 | 1 |
| Total |  |  |  |  |  | 196 |

DDDvet DDD values in mg/kg/day defined by the European Medicines Agency (EMA)

DDDjp DDD values in mg/kg/day defined in this study using DDD values of antimicrobial products approved and marketed for use in Japan

The DDD values for intramammary products for lactating cows and intrauterine products were assigned by dividing the daily dose per teat by 635 kg (standard weight of dairy cows assigned based on the average weight of dairy cows in 2014 in Japan (Livestock Improvement Association of Japan. Summary of dairy cow herd ability test results 2016. Available from: http://liaj.lin.gr.jp/japanese/newmilk/17/H28matome.pdf). The DDD values for intramammary products for dry cows were assigned by multiplying the course dose per teat by four (number of teats) and dividing it by 635 kg and an assumed long-acting factor of four days (Fujimoto K, Shimizu H, Kikuchi M, Matsui T, Ito M, Hashimoto S et al. 2020. Establishing DDD values for veterinary antimicrobial products in Japan for measuring antimicrobial use on cattle and poultry farms. Journal of Japanese Veterinary Medical Association (in press)).
