# Supplementary material for: Establishing defined daily doses (DDDs) for antimicrobial agents used in pigs, cattle and poultry in Japan and comparing them with European DDD values
Source: PLoS One. 2021 Apr 16;16(4):e0245105. doi: 10.1371/journal.pone.0245105 (PMC8051781; doi:10.1371/journal.pone.0245105)
Supplement: S3 Table — (DOCX) [file pone.0245105.s003.docx]

**S3 Table**

**Japanese DDD values (DDDjp) defined in this study for antimicrobial agents used in poultry in Japan and corresponding DDD values (DDDvet) defined by the European Medicines Agency**

| Antimicrobial class | Antimicrobial agent  (active ingredient) | Product type | Administration route | DDDvet  (mg/kg) | DDDjp  (mg/kg) | Number of products |
| --- | --- | --- | --- | --- | --- | --- |
| Tetracyclines | Oxytetracycline | Single substance | Injection |  | 31.3 | 5 |
| Aminoglycosides | Dihydrostreptomycin | Single substance | Injection |  | 62.5 | 2 |
|  | Kanamycin | Single substance | Injection |  | 37.5 | 11 |
| Tetracyclines | Doxycycline | Single substance | Oral | 15.0 | 17.3 | 12 |
|  | Chlortetracycline | Single substance | Oral | 30.0 | 35.1 | 6 |
|  | Oxytetracycline | Single substance | Oral | 39.0 | 40.7 | 7 |
| Amphenicoles | Thianphenicol | Single substance | Oral | 55.0 | 39.0 | 7 |
|  | Florfenicol | Single substance | Oral | 30.0 | 20.0 | 1 |
| Penicillins | Amoxicillin | Single substance | Oral | 16.0 | 30.0 | 8 |
|  | Ampicillin | Single substance | Oral | 108.0 | 16.3 | 8 |
|  | Procaine benzylpenicillin | Combination | Oral |  | 4.7 | 5 |
| Sulfonamides | Sulfadimethoxine | Single substance | Oral | 65.0 | 106.3 | 2 |
|  | Sulfadimethoxine | Combination | Oral | 31.0 | 56.2 | 2 |
|  | Sulfamonomethoxine | Single substance | Oral |  | 97.5 | 7 |
|  | Sulfamonomethoxine | Combination | Oral | 9.5 | 31.9 | 4 |
|  | Sulfamethoxazole | Combination | Oral | 27.0 | 32.5 | 2 |
| Trimethoprims | Trimethoprim | Combination | Oral | 6.4 | 6.4 | 4 |
|  | Ormethoprim | Combination | Oral |  | 10.6 | 4 |
| Macrolides | Tylosin | Single substance | Oral | 81.0 | 70.5 | 13 |
|  | Tylvalosin | Single substance | Oral | 25.0 | 47.6 | 3 |
| Lincosamides | Lincomycin | Single substance | Oral | 8.6 | 5.1 | 6 |
| Aminoglycosides | Streptomycin | Combination | Oral |  | 23.4 | 3 |
|  | Kanamycin | Combination | Oral |  | 23.4 | 2 |
| Fluoroquinolones | Norfloxacin | Single substance | Oral |  | 20.0 | 1 |
|  | Enrofloxacin | Single substance | Oral | 10.0 | 11.5 | 1 |
|  | Ofloxacin | Single substance | Oral |  | 7.5 | 1 |
| Other quinolones | Oxolinic acid | Single substance | Oral | 20.0 | 39.7 | 4 |
| Total |  |  |  |  |  | 131 |

DDDvet DDD values in mg/kg/day defined by the European Medicines Agency (EMA)

DDDjp DDD values in mg/kg/day defined in this study using DDD values of antimicrobial products approved and marketed for use in Japan
